# A225 UPTAKE AND OUTCOMES OF MENTAL HEALTH AND NUTRITION COUNSELLING FOR RECENTLY DIAGNOSED IBD PATIENTS.

**DOI:** 10.1093/jcag/gwae059.225

**Published:** 2025-02-10

**Authors:** M Narous, G Ly, K vagianos, M Kredentser, L Graff, S Shaffer, H Singh, C N Bernstein

**Affiliations:** University of Manitoba Max Rady College of Medicine, Winnipeg, MB, Canada; University of Manitoba Max Rady College of Medicine, Winnipeg, MB, Canada; University of Manitoba Max Rady College of Medicine, Winnipeg, MB, Canada; University of Manitoba Max Rady College of Medicine, Winnipeg, MB, Canada; University of Manitoba Max Rady College of Medicine, Winnipeg, MB, Canada; University of Manitoba Max Rady College of Medicine, Winnipeg, MB, Canada; University of Manitoba Max Rady College of Medicine, Winnipeg, MB, Canada; University of Manitoba Max Rady College of Medicine, Winnipeg, MB, Canada

## Abstract

**Background:**

Inflammatory bowel disease (IBD) significantly impacts both physical and mental health, with patients experiencing elevated levels of anxiety and depression. Nutritional management is also crucial, as dietary choices can influence physical symptoms. Despite the importance of integrated mental health and nutrition services, numerous barriers, such as limited awareness and access, contribute to suboptimal engagement among patients with IBD.

**Aims:**

We sought to assess the uptake of integrated psychologist and dietician services in a clinical setting, and to further understand the mental health and nutrition needs of individuals with IBD.

**Methods:**

In a pilot study initiated in May 2022, recruiting from an IBD clinic at Manitoba’s provincial tertiary hospital, consecutive individuals ages 16+ with IBD diagnosed within 3 years were invited to enrol. Participants completed baseline screening tools including the DASS-21, PHQ8, GAD7, PROMIS, and SaskIBD (nutrition risk) and were offered appointments with an IBD psychologist, dietitian or both, when flagged through the screening tools. Descriptive data were collected regarding clinical screening, psychological diagnoses and interventions, as well as dietary needs and nutrition counselling provided.

**Results:**

Of 226 eligible, 101 were enrolled (42% Crohn’s disease), and 64 accepted referral to clinical psychology. Of those, >50% and 36-49% met screening criteria for moderate to severe depression and anxiety, respectively. Median scores (± std dev) for DASS-21 were 14 ±9.6 (depression), 8 ±6.6 (anxiety), and 16 ±7.9 (stress); PHQ8 10 ±5.2; and GAD7 8 ±4.7. At an initial semi-structured diagnostic interview, approximately 50% of patients were confirmed to have a psychological diagnosis, most commonly depression (20.6%), anxiety (31.7%) and insomnia/sleep disorder (15.9%). Median number of psychologist visits was 1 ±4.13 (range 0-19); 44.4% of patients returned for follow-up. Of the 37 patients who were not flagged/declined to proceed with the psychologist, only 4 screened positive (1 moderate depression; 3 moderate-severe anxiety). Almost all participants (n=84) were flagged to see the dietitian, with the main needs relating to types of food to avoid and strategies for weight gain or loss. Of those, 52% screened positive for moderate to severe risk of malnutrition, with a median SaskIBD score of 3 ±2.1.

**Conclusions:**

In a prospective cohort of recently diagnosed IBD patients, there is a demonstrable need for integrated mental health and dietitian expertise with excellent uptake of these services when easily accessible in an outpatient gastroenterology clinic. Ongoing data are being collected to characterize outcomes of this comprehensive model of care for IBD patients.

**Funding Agencies:**

CCC

